# Medication Extraction and Drug Interaction Chatbot: Generative Pretrained Transformer-Powered Chatbot for Drug-Drug Interaction

**DOI:** 10.1016/j.mcpdig.2024.09.001

**Published:** 2024-10-09

**Authors:** Won Tae Kim, Jaegwang Shin, In-Sang Yoo, Jae-Woo Lee, Hyun Jeong Jeon, Hyo-Sun Yoo, Yongwhan Kim, Jeong-Min Jo, ShinJi Hwang, Woo-Jeong Lee, Seung Park, Yong-June Kim

**Affiliations:** a Department of Urology, Chungbuk National University Hospital, Cheongju, Republic of Korea; b Department of Biomedical Engineering, Chungbuk National University Hospital, Cheongju, Republic of Korea; c Department of Family Medicine, Chungbuk National University Hospital, Cheongju, Republic of Korea; d Department of Internal Medicine, Chungbuk National University Hospital, Cheongju, Republic of Korea; e Department of Urology, College of Medicine, Chungbuk National University, Cheongju, Republic of Korea; f Department of Biomedical Engineering, College of Medicine, Chungbuk National University, Cheongju, Republic of Korea; g Department of Family Medicine, College of Medicine, Chungbuk National University, Cheongju, Republic of Korea; h Department of Internal Medicine, College of Medicine, Chungbuk National University, Cheongju, Republic of Korea

## Abstract

**Objective:**

To assist individuals, particularly cancer patients or those with complex comorbidities, in quickly identifying potentially contraindicated medications when taking multiple drugs simultaneously.

**Patients and Methods:**

In this study, we introduce the Medication Extraction and Drug Interaction Chatbot (MEDIC), an artificial intelligence system that integrates optical character recognition and Chat generative pretrained transformer through the Langchain framework. Medication Extraction and Drug Interaction Chatbot starts by receiving 2 drug bag images from the patient. It uses optical character recognition and text similarity techniques to extract drug names from the images. The extracted drug names are then processed through Chat generative pretrained transformer and Langchain to provide the user with information about drug contraindications. The MEDIC responds to the user with clear and concise sentences to ensure the information is easily understandable. This research was conducted from July 1, 2022 to April 30, 2024.

**Results:**

This streamlined process enhances the accuracy of drug-drug interaction detection, providing a crucial tool for health care professionals and patients to improve medication safety. The proposed system was validated through rigorous evaluation using real-world data, reporting high accuracy in drug-drug interaction identification and highlighting its potential to benefit medication management practices considerably.

**Conclusion:**

By implementing MEDIC, contraindicated medications can be identified using only medication packaging, and users can be alerted to potential drug adverse effects, thereby contributing to advancements in patient care in clinical settings.

Drug therapy plays a critical role in medicine and relies on the sciences of pharmacology and pharmacy for continuous advancements and proper management. Since the early 20th century, the development of modern medicines has considerably reduced mortality rates caused by fatal diseases. This advancement has led to aging populations in some countries, resulting in increased use of medication among the elderly to manage diseases and maintain their health. Elderly patients with cancer often experience polypharmacy, which is defined as the use of 5 or more drugs. Elderly cancer patients are exposed to polypharmacy at rates ranging from 11% to 96%, depending on the stage of prevalence.^1^

Simultaneously taking multiple medications increases the risk of unexpected reactions owing to drug-drug interactions (DDIs), which can lead to negative effects of the drugs.^2^ These DDIs can reduce the therapeutic effects of the drugs, cause adverse effects, frailty, falls, cognitive impairments, and severe toxicity, and even lead to hospitalization or death, thereby posing severe safety concerns.^3^, ^4^, ^5^, ^6^ In the United States, adverse DDIs result in an average of 74,000 emergency room visits and 195,000 hospitalizations annually.^7^ According to the Centers for Disease Control and Prevention, approximately 10% of Americans take at least 5 medications concurrently, whereas 36% over 60 years of age take 5 or more drugs regularly, with ∼15% of these medications posing DDI risks.^8^

Emphasis is placed on predicting DDIs to prevent negative outcomes resulting from the use of such medications. Ip et al^9^ reported that hospital readmission rates could decrease by 27% with appropriate medication monitoring. However, ordinary people tend to have negative perceptions regarding spending substantial time on monitoring, such as revisiting hospitals or contacting health care providers.

Thus, there is a need for a model that allows ordinary people who lack medical knowledge to verify DDIs quickly without investing many time. To address this challenge, this study presents an artificial intelligence (AI) chatbot for DDI known as Medication Extraction and Drug Interaction Chatbot (MEDIC). Patients or their families can upload photos of prescription medication packages received from hospitals to MEDIC, and the chatbot provide DDI information based on the uploaded images. The MEDIC uses optical character recognition (OCR) technology to extract medication names from medication envelope and detects DDIs among these medications using Chat generative pretrained transformer (ChatGPT). To internalize knowledge regarding DDIs in ChatGPT, we constructed a DDI database and integrated it with ChatGPT through the Langchain framework. The contributions of this study are as follows: First, a novel approach is proposed that integrates OCR, ChatGPT, and Langchain to improve DDI assessments. Second, the proposed approach was validated using real-world data, and its effectiveness for identifying potential DDIs was reported. Section II introduces the related work, which includes previous studies similar to our research. Section III outlines the proposed methods to address the issues of DDI and the roles of OCR, ChatGPT, and Langchain in resolving them. Section IV presents the experimental results to report the effectiveness of the proposed system. Section V discusses the limitations of this study and proposes means of addressing them. Finally, Section VI concludes the research and suggests future research directions.

The DDIs are known to cause unwanted adverse effects. The accurate prediction of DDIs is crucial for human health, necessitating the development of precise computational methods. Several approaches have been developed, leveraging biomedical information to advance computational strategies for DDI prediction. Gao et al^10^ proposed the AutoDDI method for predicting DDI adverse effects by automatically designing graph neural network architectures considering the molecular structure of drugs. The optimized architecture that was efficiently discovered through reinforcement learning achieved excellent performance on 2 real-world datasets. An automatic system to detect drug interactions using clinical data was implemented at Rennes Hospital.^11^ This system analyzes the interactions between drugs using OrientDb and Spark, and supports safe drug management in hospitals by predicting drug adverse effects through machine learning models. Rohani and Eslahchi^12^ proposed neural network-based method for drug-drug interaction prediction using various drug-related information to predict unknown DDIs. Zhang et al^13^ introduced a novel algorithm using a convolutional neural network (CNN) architecture known as CNN-DDI for DDI prediction. By extracting feature interactions from drug categories, targets, pathways, and enzymes as feature vectors and employing the Jaccard similarity for drug similarity measurement, a new CNN was developed as the DDI prediction model.

With the rise of scanning technology such as OCR, its application within medical systems is gaining increasing interest. In the study by Naeem and Coronato,^14^ an AI-based system was proposed to monitor the medication process considering patients’ cognitive impairments. This system combines deep learning classifiers, OCR, and barcode technology to minimize medication errors and support patients in taking the correct medication. Goodrum et al^15^ used OCR for text extraction and combined it with ClinicalBERT to compose electronic health record documents.

Chat generative pretrained transformer, which is based on generative pretrained transformer-3 with 175 billion parameters, has been trained on vast amounts of information and is used across various fields.^16^ In particular, ChatGPT exhibits impressive performance in the medical domain. Rao et al^17^ compared the responses of ChatGPT to the American College of Radiology appropriateness criteria for breast pain and breast cancer screening, showcasing its potential for radiologic decision-making. Similarly, Chen et al^18^ evaluated the effectiveness of ChatGPT in providing treatment recommendations for breast, prostate, and lung cancer that adhere to National Comprehensive Cancer Network (NCCN) guidelines. Chat generative pretrained transformer provided at least one NCCN-concordant recommendation for 98% of the prompts, although 34.3% of these also contained recommendations that were at least partially nonconcordant with NCCN guidelines. The authors concluded that although ChatGPT can provide valuable insights, its ability to offer reliable and robust cancer treatment recommendations has limitations. Chat generative pretrained transformer can effectively predict and respond to DDIs, but it may provide incomplete guidance depending on the context.^19^ We integrated the Langchain framework to address the abovementioned limitations of ChatGPT by internalizing DDI knowledge.

## Patients and Methods

### Dataset

Two types of datasets related to pharmaceutical constraints were employed in this study: the drug authorization list (DAL), and drugs contraindicated in combination (DC). The DAL dataset was obtained from drug safety country (https://nedrug.mfds.go.kr/index) in Excel format. It comprises serial numbers, product names in Korean, and product names in English, with a total of 48,432 entries. The DAL dataset was used to extract drug names from the OCR Name results. In this process, we exclusively used the product name in Korean category, thereby retaining only this category although removing the others to reduce the data processing time. The DC dataset was obtained from the public data portal (https://www.data.go.kr/index.do), also in Excel format. It comprises ingredient code A, product name A, ingredient code B, product name B, etc., with a total of 469,497 combinations. The DC dataset was employed to identify contraindications between 2 drugs and detect potentially hazardous drug combinations. The proposed method determines whether 2 drugs should be contraindicated when their names are found in the same column under product names A and B in the DC dataset. Only the product name A and product name B categories were used in this process. Therefore, we retained these 2 categories although removing the others to reduce the data processing time.

**Figure 1:**
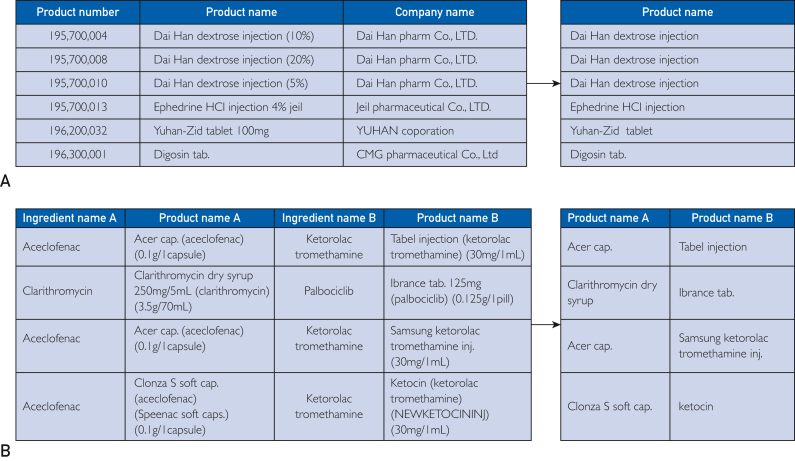
Before and after data processing of 2 datasets: (A) drug authorization list and (B) drugs contraindicated in combination.

In this study, drug contraindications were defined as scenarios in which the concurrent use of 2 or more active ingredients may impact their therapeutic effects or induce severe adverse reactions. Consequently, the identification of contraindications was based solely on the active ingredients, rendering dosage information extraneous for our purposes. To enhance the precision of the text similarity analysis, which is an essential step in identifying potential contraindications, the dosage information was systematically removed from each dataset during data preprocessing. This process led to the creation of duplicate entries. For example, Acer cap.(0.1g) and Acer cap.(0.2g) were both transformed into Acer cap. after removing the dosage information. Consequently, these duplicate entries were reduced to a single entry. As a result of this preprocessing, the number of entries in the DAL dataset decreased from 48,432 to 38,660, and the number of entries in the DC dataset decreased from 469,497 to 162,876. The outcomes of this preprocessing are illustrated in Figure 1.

This study was performed with strict adherence to international ethical standards in both research design and methodology. The data used in this research were sourced from publicly available datasets that do not contain personally identifiable or sensitive information. Consequently, the study does not involve human patients, and approval from an institutional review board or ethical committee was not required.

### Proposed Model

This section introduces the MEDIC system that combines OCR and ChatGPT (OpenAI, USA). Medication Extraction and Drug Interaction Chatbot employs OCR technology and a text similarity evaluation algorithm to extract drug names from drug bag images. In this process, we performed experiments by intentionally damaging the drug bags to simulate real-world conditions. Subsequently, it uses ChatGPT to analyze the extracted drug names and identify potentially hazardous drug combinations. Recognizing the limitations of ChatGPT in terms of drug information, we integrated the Langchain framework and DC dataset to ensure that users receive high-quality responses.

**Figure 2:**
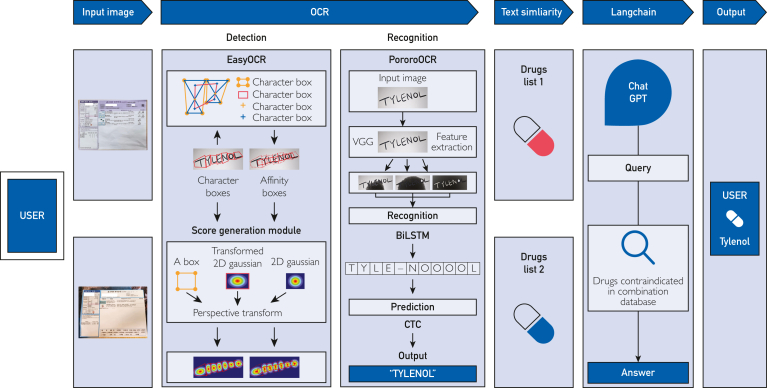
System architecture of MEDIC, which consists of OCR, text similarity, and the Langchain framework. 2D, two dimension; BiLSTM, bidirectional long short-term memory; ChatGPT, Chat generative pretrained transformer; CTC, connectionist temporal classification; MEDIC, Medication Extraction and Drug Interaction Chatbot; OCR, optical character recognition; VGG, visual geometry group.

As depicted in Figure 2, the operational flow of MEDIC begins by receiving 2 images of drug bags from a user. The reason for receiving images of 2 drug bags is that, although a single hospital may prescribe medication after considering DDIs, it is anticipated that another hospital may not account for medications prescribed elsewhere unless explicitly informed by the patient. Therefore, combinations are only formulated from medications found in different bags and not from those found within a single bag. For example, assume medications A_1, A_2, and A_3 are from company A’s drug bag, and B_1, B_2, and B_3 are from company B’s drug bag. In this case, MEDIC will not form combinations such as A_1 and A_2 from a single drug bag but will instead create combinations from medications found in different drug bags, such as A_1 and B_1 or A_1 and B_2. In this progress, it then uses EasyOCR to locate the text within the images and PororoOCR for text recognition. Using text similarity analysis and the DAL dataset, the system extracts only drug names using the OCR results and passes them to ChatGPT.

Chat generative pretrained transformer, in conjunction with the Langchain framework and a prebuilt database using the DC dataset, formulates queries for the database and retrieves only relevant data. Subsequently, the system determines whether there are any contraindications among the drug names extracted from the medication packaging images and provides the results to the user.

**Figure 3:**
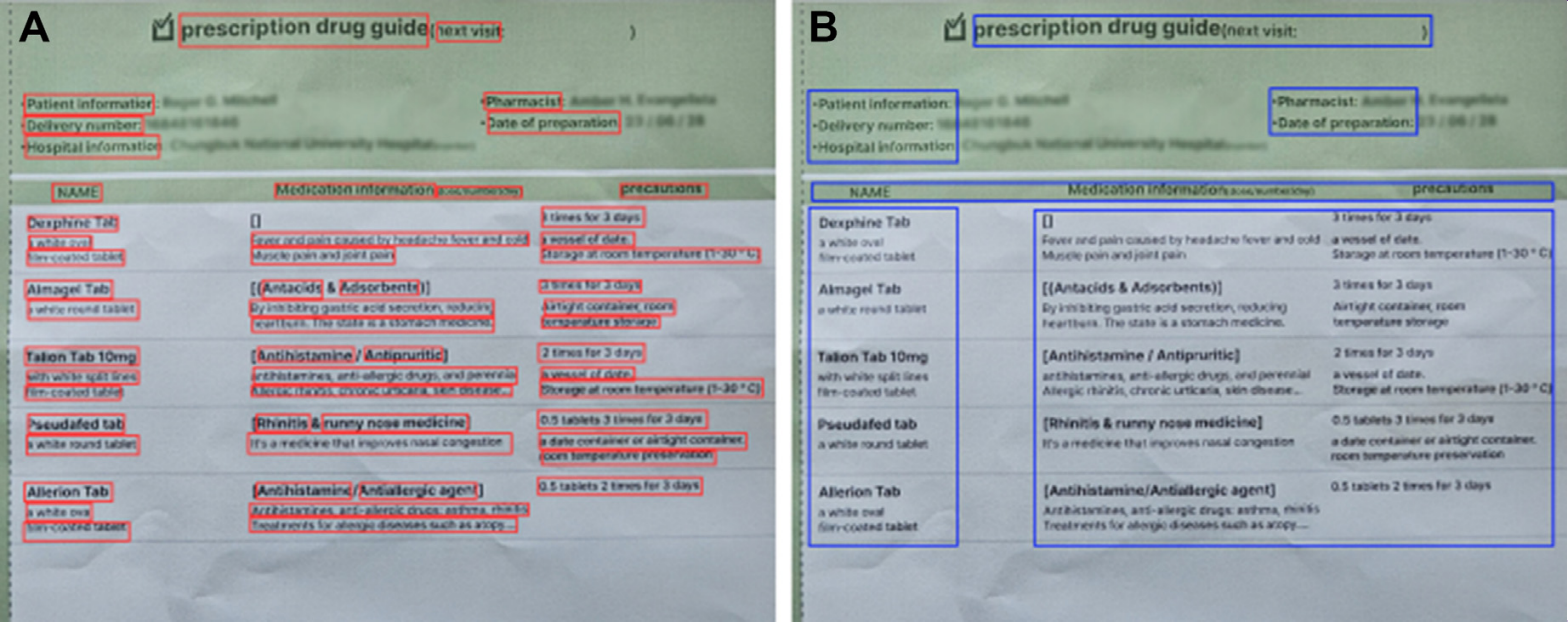
Bounding box results obtained by Craft technology: (A) EasyOCR and (B) PororoOCR.

The first reason that we used 2 types of OCR is owing to the technology of EasyOCR Craft. The Craft technology predicts the region scores indicating the position of characters and affinity scores indicating the distance between characters, enabling the detection of word positions.^20^ As shown in Figure 3, EasyOCR employs a more detailed implementation of Craft technology than PororoOCR, placing bounding boxes around each word individually. Thus, although EasyOCR extracts words one by one, PororoOCR extracts all words within the bounding box simultaneously. Medication Extraction and Drug Interaction Chatbot uses the EasyOCR Craft technology and text similarity analysis to extract only drug names from the OCR results efficiently. The second reason is the recognition rate of PororoOCR. PororoOCR has a structure similar to EasyOCR. However, unlike EasyOCR, PororoOCR is a model that is specialized in Korean and English, having been additionally trained on Korean text images provided by AIhub and internal data from KaKaoBrain. The test results using 30 randomly selected prescription bags written in Korean found that PororoOCR identified 2-3 more drug names accurately than EasyOCR when using text similarity. The best performance was achieved when the text similarity threshold was set to 0.7.

The large language model (LLM) of MEDIC uses the gpt-3.5-turbo-16k-0613 model from the ChatGPT series. Although ChatGPT, as described by Juhi et al,^19^ can predict potential DDIs, ChatGPT may sometimes provide incomplete guidelines depending on the context. To address this limitation, this study adopted the Langchain framework method to complement the provision of incomplete guidelines by ChatGPT. Contraindication information was constructed using the DC dataset by MySQL and the Langchain framework connected ChatGPT with MySQL to deliver contraindication information regarding the OCR results to ChatGPT. This approach effectively addresses the limitations of incomplete guidelines in ChatGPT by providing supplementary contraindication information.

## Results

To validate the effectiveness of the proposed system, we evaluated the performance of both the OCR and overall model. The OCR performance was measured using 3 metrics: accuracy, character error rate (CER), and word error rate (WER).^21^ The performance of the overall model was assessed based on its ability to distinguish between contraindications in 2 types of medication packages.

The OCR accuracy was considered to be one when all 8 drug names were precisely extracted from the medicine package; this was achieved with an accuracy of 0.125 per drug name. The CER was calculated using the total length of the drug names (N) and number of incorrectly deleted (D), substituted (S), and inserted (I) syllables, as follows:

(1) CER=(S+D+I)÷N.

The WER was calculated using the number of drug names per bag (N) and number of incorrectly deleted (D), substituted (S), and added (I) words, as follows:

(2) WER=(S+D+I)÷N.

The OCR performance was evaluated using 150 data samples (1,200 words), as listed in Table. The proposed OCR model identified 1,198 words, among which 1,190 were accurately recognized. Consequently, it exhibited an accuracy of 99.17%, a WER of 0.83%, and a CER of 1.55%.

**Table:** Performance Results of OCR Using Text Similarity for 150 Drug Packages

Total words	OCR result	Correct words
1200	1198	1190
Accuracy	WER	CER
99.17%	0.83%	1.55%

CER, character error rate; OCR, optical character recognition; WER, word error rate.

The overall system performance was assessed using the accuracy metric. We prepared 3 types of medication packages, designated as A, B, and C. Package A included 3 drugs from the DC dataset and 5 not included in the dataset. Package B contained 3 drugs that are contraindicated when used with those in Package A, along with 5 additional drugs that were not included in the DC dataset. Package C comprised 8 drugs that were not present in the DC dataset. In addition, the drug packaging in packages A, B, and C were prepared in 5 different fonts with 10 instances each, resulting in 50 instances for each package for a total of 150 data samples.

**Figure 4:**
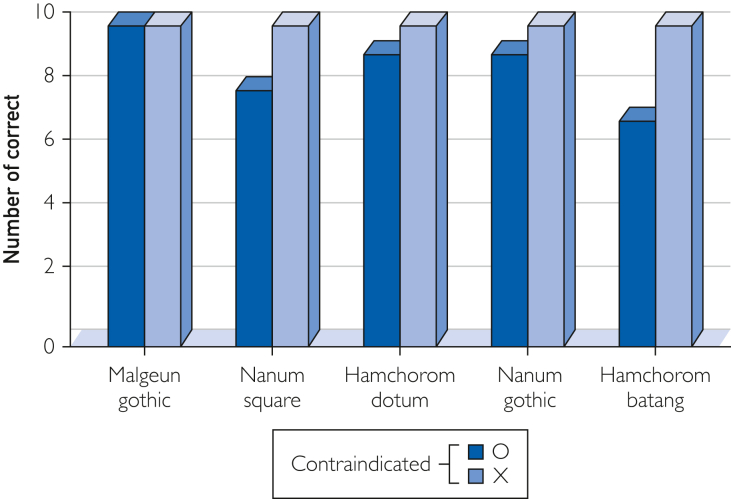
Evaluation results of DDI presence by font type. DDI, drug-drug interaction.

The experimental method involved inputting combinations of Package A and Package B or Package A and Package C into MEDIC. For the A and B combination, we defined the correct response as identifying all possible combinations between A and B, then identifying all contraindications, and responding with specific contraindications, such as A_1 and B_2 are contraindicated. Similarly, for the A and C combination, we defined the correct response as identifying all possible combinations between A and C, then checking for all contraindications, and responding with no contraindications exist if there are none. As shown in Figure 4, MEDIC provided accurate responses for 93 out of 100 combinations.

## Discussion

This study aimed to address the crucial issue of identifying potentially hazardous drug combinations from drug packaging images by developing the MEDIC system, which combines OCR and ChatGPT. The research results were evaluated based on the OCR performance metrics accuracy, CER, and WER. The existing OCR models mostly failed to accurately extract drug names. When character similarity was used, EasyOCR found about a fourfold improvement, PororoOCR about a threefold improvement, and the model used in our research about a twofold improvement. In addition, we focused on enhancing the performance of ChatGPT. According to Juhi et al,^23^ ChatGPT can effectively predict and respond to DDIs, but it may provide incomplete guidance in certain situations. We concluded that this issue is due to the hallucination phenomenon of LLM models. By using Langchain, we improved performance, and as a result, the original ChatGPT correctly identified 25 out of 100 contraindicated combinations, whereas the ChatGPT model enhanced with Langchain correctly identified 99.

These results suggest that MEDIC can potentially reduce emergency room visits and hospitalizations for polypharmacy patients by providing information on clinically contraindicated drug combinations in a format such as Drug A and Drug B should not be co-administered. Instead of offering specific instructions on what actions to take, MEDIC encourages patients to discontinue only harmful medications. In addition, MEDIC emphasizes ease of use by allowing users to receive answers simply by taking a photo of their medication bag, with responses provided in an easy-to-understand format.

However, despite the overall success of the MEDIC system, several considerations need to be addressed. First, although the OCR accuracy is high, the use of both the EasyOCR and PororoOCR models increases the data processing time. This could potentially lead to delayed response times in situations requiring real-time data processing. Second, the efficiency of the system relies heavily on the accuracy of the underlying drug contraindication database. Although the integration of the Langchain framework and DC dataset addresses the medical knowledge limitations of ChatGPT, continuous database updates and validation by medical experts are essential to ensure system reliability and accuracy. Finally, cost-related issues exist. As MEDIC uses the LLM model through the ChatGPT application programming interface, the structure entails increasing the monetary costs as more users utilize the system.

In future research, there is a need to develop a new single OCR model that combines the intricate Craft technology of EasyOCR with the language-specific recognition capabilities of PororoOCR. Using a single OCR model would reduce the data processing time, thereby enhancing the efficiency even in clinical settings. In addition, to address cost-related issues, the development of a small language model^22^ focused on specific fields instead of an LLM is required. This approach could reduce the costs by minimizing the required computer resources and training data while maintaining effectiveness.

## Conclusion

By integrating OCR technology and ChatGPT, the proposed MEDIC system exhibits potential for the identification and management of DDIs, particularly in the context of polypharmacy among elderly patients. Medication Extraction and Drug Interaction Chatbot achieved high accuracy in extracting drug names from medication packaging and identifying potential DDIs, thereby addressing this critical requirement in modern health care. The successful application of this system in a real-world scenario highlights its potential for enhancing medication safety and patient care. Future work should focus on expanding the database to include a broader range of medications and improving the ability of the system to learn from new data, ensuring that it remains a valuable resource in the ever-evolving health care field.

## Potential Competing Interests

The authors report no competing interests.

## Ethics Statement

This study was conducted with full adherence to ethical guidelines in both the research design and experimental processes. The data used in this study were collected from publicly accessible sources, such as public datasets, and do not involve any personally identifiable or sensitive information. Therefore, this research does not involve human subjects or privacy-related issues, and approval from an institutional review board or ethical board was not required. The researchers have followed international ethical guidelines throughout the study and have ensured transparency and ethical responsibility in the conduct of the research.
